# Efficacy of laryngeal mask airway against postoperative pharyngolaryngeal complications following thyroid surgery: a systematic review and meta-analysis of randomized controlled studies

**DOI:** 10.1038/s41598-022-21989-5

**Published:** 2022-10-28

**Authors:** Kuo-Chuan Hung, Shao-Chun Wu, Chih-Wei Hsu, Ching-Chung Ko, Jui-Yi Chen, Ping-Wen Huang, I.-Wen Chen, Cheuk-Kwan Sun

**Affiliations:** 1 Department of Anesthesiology, Chi Mei Medical Center, Tainan, Taiwan; 2 Department of Anesthesiology, Kaohsiung Chang Gung Memorial Hospital, Chang Gung University College of Medicine, Kaohsiung, Taiwan; 3 Department of Psychiatry, Kaohsiung Chang Gung Memorial Hospital and Chang Gung University College of Medicine, Kaohsiung, Taiwan; 4 Department of Medical Imaging, Chi Mei Medical Center, Tainan, Taiwan; 5 Institute of Biomedical Sciences, National Sun Yat-Sen University, Kaohsiung, Taiwan; 6 Department of Health and Nutrition, ChiaNai University of Pharmacy and Science, Nanjing, China; 7 Division of Nephrology, Department of Internal Medicine, Chi Mei Medical Center, Tainan, Taiwan; 8 Department of Emergency Medicine, Show Chwan Memorial Hospital, Changhua, Taiwan; 9 Department of Anesthesiology, Chi Mei Medical Center, Liouying, Tainan, Taiwan; 10 Department of Emergency Medicine, E-Da Hospital, No.1, Yida Road, Jiaosu Village, Yanchao District, Kaohsiung, 82445 Taiwan; 11 College of Medicine, I-Shou University, Kaohsiung, Taiwan

**Keywords:** Health care, Medical research

## Abstract

This meta-analysis aimed at investigating the effectiveness of laryngeal mask airway (LMA) against postoperative pharyngolaryngeal complications after thyroidectomy. MEDLINE, Cochrane Library, google scholar, and EMBASE databases were searched from inception through February, 2021, for randomized controlled trials (RCTs) comparing the incidence of pharyngolaryngeal complications following the use of LMA or endotracheal tube (ETT). Pooled results from seven RCTs involving 600 patients showed an association of LMA with a reduced risk of postoperative sore throat (POST) at 24 h [risk ratio (RR) 0.75, *p* = 0.006, four trials], but not at 1 h and 48 h after thyroidectomy. POST severity and hoarseness risk were lower in the LMA group than the ETT group at 1 h, 24 h, and 48 h (all *p* < 0.05). Nevertheless, hoarseness severity was lower in the LMA group only at postsurgical 48 h [standardized mean difference = − 0.35, *p* = 0.008, three trials]. Moreover, the risk of emergence cough was lower in patients using LMA than those receiving ETT (RR = 0.14, *p* = 0.002, two trials). The two groups did not differ in the severity of dysphagia at postoperative 1 h, 24 h, and 48 h. This meta-analysis showed that LMA may be associated with fewer pharyngolaryngeal complications compared to ETT without airway impacts. The limited number of included studies warrants further research to support our findings.

## Introduction

Postoperative sore throat (POST), which is a frequent complication following anesthesia with endotracheal tube (ETT), affects up to 62% of patients^1, 2^. It has been reported to be the eighth most common postoperative adverse event and could last for 2–3 days^3^, causing dissatisfaction and discomfort as well as delaying patients’ resumption of normal daily activities^4^. The incidence of POST is particularly high (68.4%) in patients receiving thyroid surgery^5–7^ for a variety of thyroid diseases, which have a higher prevalence in the female population^8^. The seriousness of the problem is reflected by the high proportion of patients undergoing thyroid surgery in the ambulatory care and overnight stay settings^9^.

The laryngeal mask airway (LMA), which allows the maintenance of upper airway patency without the need for direct visualization of the vocal cords and could avoid potential trauma during the tracheal intubation process, is an alternative to ETT^10^ that is gaining popularity in head and neck surgery in the last two decades^11–14^. The potential beneficial impacts of using LMA on minimizing potential damage to the vocal cords and preventing postoperative pharyngolaryngeal symptoms^15^ have been supported by the results of previous studies showing a significant reduction in the incidences of postoperative hoarseness and sore throat in patients using LMA compared to those receiving ETT for general anesthesia^16, 17^. Indeed, a large-scale study recruiting 5264 patients subjected to non-thyroid surgery under general anesthesia revealed an incidence of POST up to 45.4% in those receiving ETT compared to 17.7% in those using LMA^1^.

Focusing on thyroid surgery, several previous studies have also demonstrated the feasibility and safety of LMA in this patient population^9, 18^. Nevertheless, the efficacy of LMA against postoperative pharyngolaryngeal complications was not investigated in a systematic approach. Therefore, the present meta-analysis aimed at comparing the risks of postoperative pharyngolaryngeal symptoms between ETT and LMA in patients receiving thyroidectomy under general anesthesia. We present the following article in accordance with the PRISMA reporting checklist.

## Methods

This meta-analysis was reported according to the recommendations of the PRISMA statement and was registered with the International Prospective Register of Systematic Reviews (CRD42021248665).

### Eligibility criteria

Studies that investigated the incidence of postoperative pharyngolaryngeal complications associated with the use of LMA in patients receiving thyroid surgery were considered eligible if they fit into the predefined criteria : (a) Patient population: adults undergoing thyroid surgeries (including those combined with parathyroid procedures), (b) Intervention: use of LMA (regardless of manufacturers), (c) Comparison: the use of ETT, (d) Outcomes: risk of postoperative pharyngolaryngeal complications or airway-related outcomes. No restrictions were placed on language, sample size, and publication date. Studies that were (1) not randomized controlled trials (RCTs); (2) not published in peer-reviewed journals or those published only as abstracts or letters were excluded.

### Information sources and search strategy

A systematic literature search was executed using MEDLINE, Cochrane CENTRAL register of controlled trials, Embase, and Google scholar databases from their inception dates till February 17, 2022. The following keywords and medical subject headings was used: ("Thyroid surger*" or "Thyroidectom*" or "Parathyroid surgery" or "Thyroid" or "goiter" or "Parathyroid") and ("LMA" or "Laryngeal mask airway*" or "Laryngeal Mask" or "supraglottic airway device*" or "supraglottic"). The search strategy for one of the databases are shown in Table 1.

**Table 1:** Search strategies for Medline.

1	("Thyroid surger*" or "Thyroidectom*" or "Parathyroid surgery" or "Thyroid" or "goiter" or "Parathyroid").mp
2	exp "Endocrine Surgical Procedures"/
3	("LMA" or "Laryngeal mask airway*" or "Laryngeal Mask" or "supraglottic airway device*" or "supraglottic").mp
4	exp "Laryngeal Masks"/ or exp "Masks"/
5	(1 or 2) and (3 or 4)
6	7 and (((randomized controlled trial or controlled clinical trial).pt. or randomi*ed.ab. or placebo.ab. or drug therapy.fs. or randomly.ab. or trial.ab. or groups.ab.) not (exp animals/ not humans.sh.))

### Selection process and data collection

After excluding unsuitable articles by independent screening of their titles and abstracts, two researchers scrutinized the full-texts of the remaining reports for eligibility. Any disagreements were settled through discussion. Information retrieved from each study included first author, study setting, publication year, sample size, patient characteristics (e.g., gender), and airway-related complications. Disagreements were resolved by consultation with a third author.

### Outcomes and definitions of data items

The primary outcomes were the risk of POST and hoarseness, while the secondary outcomes consisted of severity of POST and hoarseness, changes in intraoperative airway pressure, severity of dysphagia, and risk of emergence cough.

### Risks of bias assessment

A quality assessment was independently performed by two authors who employed the Cochrane's tool (RoB 2)^19^ to evaluate the possibility of biases (i.e., allocation, performance, measurement, attrition and reporting biases). In case of disagreements, consensus was reached through discussion with the corresponding authors.

### Effect measures and data synthesis

The Review Manager 5.4 statistical software (Cochrane Collaboration) was used for the current meta-analysis. The effect sizes of continuous variables are expressed as mean difference (MD) or standardized mean difference (SMD) with 95% confidence intervals (CIs), while those of dichotomous data are presented as relative risks (RRs) and 95% CIs^20, 21^. We contacted the authors who reported outcomes as median and interquartile range in an attempt to obtain the mean and standard deviation. If there was no response to three of our emails, we proceeded with estimation of the mean and standard deviation according to the method previously described^22^. When there were less than two comparisons, results are expressed descriptively. The I^2^ statistics were used to assess heterogeneity across the included studies with significance predefined at I^2^ > 50%^23^. For a specific outcome described in 10 or more studies, the possibility of publication bias was evaluated through visual inspection of a funnel plot. Potential impact from the result of an individual trial on the overall finding was evaluated with sensitivity analysis (i.e., leave-one-out approach)^24, 25^. Two-tailed tests were conducted on all comparisons. A p-value < 0.05 was considered statistically significant.

## Results

### Study selection, study characteristics, and risk of bias assessment

**Figure 1:**
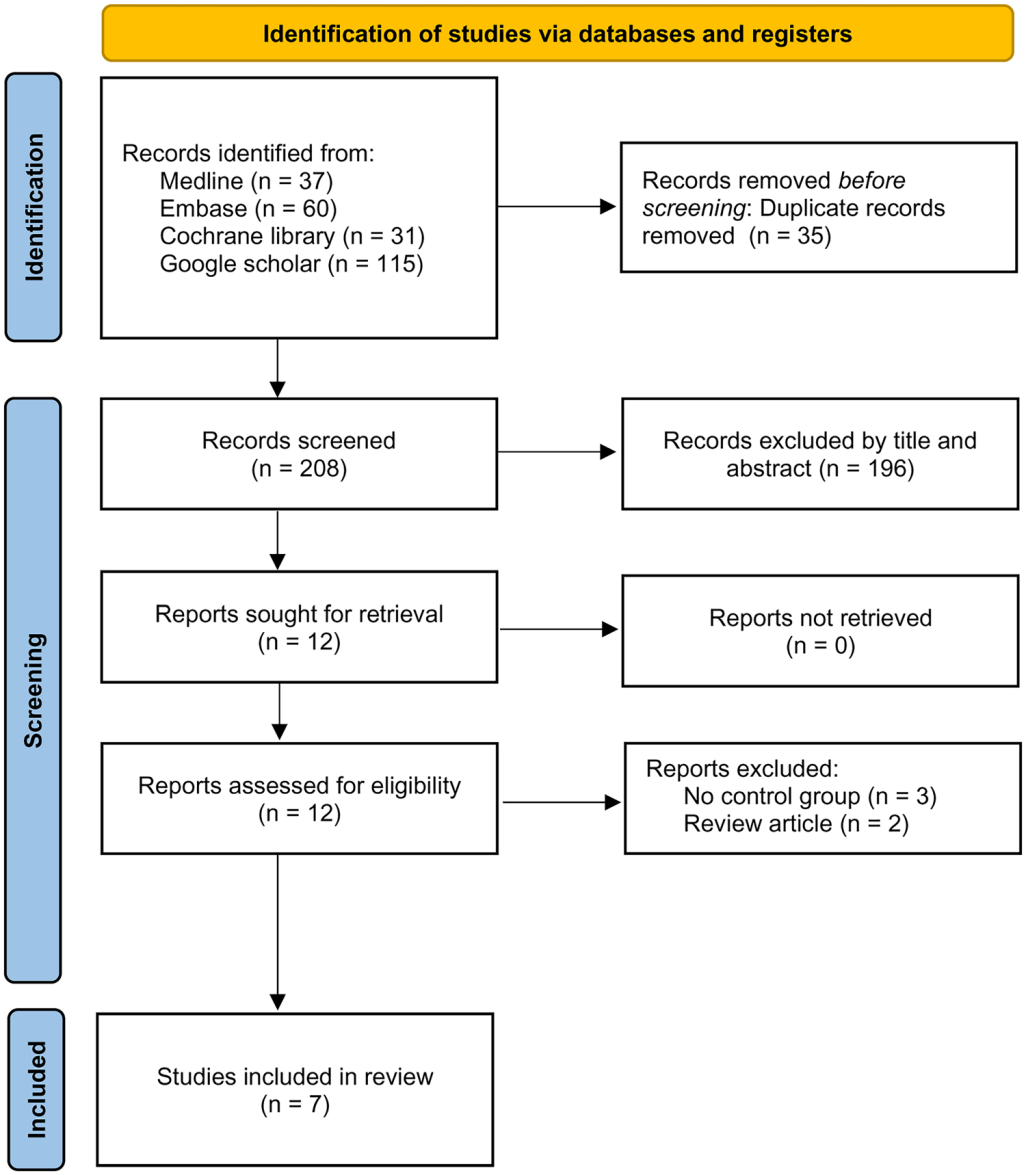
PRISMA flow diagram of study selection for the current meta-analysis.

We identified 243 potentially eligible records. After the exclusion of duplicated records (n = 35) and those which did not meet the inclusion criteria by title and abstract (n = 196), 12 studies with full-texts available were reviewed. Finally, seven trials involving 600 patients published from 2014 to 2022 were included for the current meta-analysis^9, 12, 13, 18, 26–28^. A flow diagram summarizing the process of study selection is demonstrated in Fig. 1.

The characteristics of the included studies are shown in Table 2. The median or mean age of participants ranged from 40 to 51 years with a proportion of females ranging from 66.2 to 84.6%. Six studies recruited patients receiving only thyroid surgery^9, 12, 13, 26–28^, while one trial enrolled those undergoing both thyroidectomy and parathyroidectomy^18^. The surgical time ranged from 50 to 130 min, but one trial^12^ did not provide this information. Cuff pressure of ETT and LMA were strictly controlled in five studies^9, 13, 26–28^, whereas two trials did not provide this information^12, 18^. The seven included trials were conducted in three countries. Four trials were conducted in China^9, 26–28^, two in Korea^12, 13^, and one in Greece^18^.

**Table 2:** Characteristics of studies (n = 7).

Studies	Age (years)^a^	N	Female (%)	Procedures	Surgical time^a^	Outcomes	POST severity	Country
Chun 2015	47 vs. 46	31 vs. 33	69.80	Thyroid lobectomy	NA	Acoustic analyses were performed preoperatively and at 48 h and 2 weeks postoperatively	LPS 0–10	Korea
Gong 2020a	43 vs. 43	67 vs. 65	79.50	Radical thyroidectomy	50 vs. 54	Intraoperative ventilation leak volume, peak airway pressure, and partial pressure of end-tidal carbon dioxide	NA	China
Gong 2020b	43 vs. 42	45 vs. 45	81.10	Radical thyroidectomy	54 vs. 48	Sore throat, numbness and hoarseness at 1, 24, and 48 h after surgery	VAS 0–10	China
Kotsovolis 2019	51 vs. 49	38 vs. 40	84.60	Total thyroidectomy and parathyroidectomy	105 vs. 130	Dysphagia, pharyngodynia, and incisional pain at 1, 6, 12, and 24 h after surgery. Secondary outcomes were the frequency of rescue analgesia (paracetamol) consumption and emergence cough	NRS 0–10	Greece
Ryu 2014	49 vs. 47^b^	36 vs. 37	82.20	Total thyroidectomy	105 vs. 105^b^	Sore throat, hoarseness, dysphagia, and cough were assessed at 1, 24, and 48 h after surgery	NRS 0–100	Korea
Liao 2021	40 vs. 40	35 vs. 30	66.20	Endoscopic thyroid surgery	54 vs. 57	Time to establishment of artificial airway and success rate, hemodynamics, oxygen saturation, peak airway pressure and adverse effects	NA	China
Ning 2022	45 vs. 46	49 vs. 49	76.5	Radical thyroidectomy	80 vs. 79	Primary outcome: Incidence of sore throat Secondary outcomes: Severity of sore throat; the incidence and severity of hoarseness	VAS 0–10	China

*POST* postoperative sore throat, *VAS* visual analog scale, *NRS* numerical rating scale, *LPS* laryngopharyngeal symptom score, *NA* not available.

^a^Presented as mean; ^b^presented as median.

**Figure 2:**
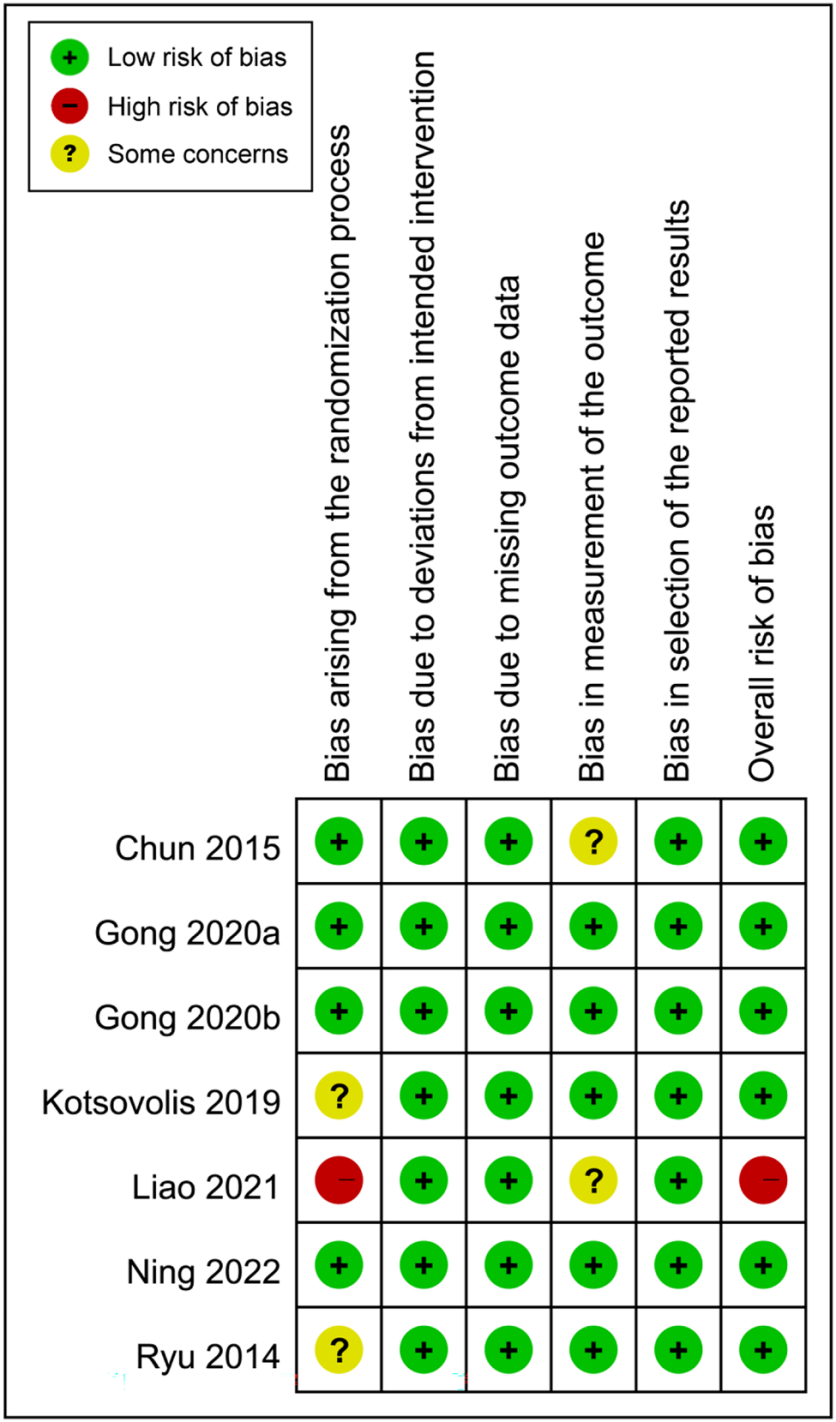
Risks of bias of the included studies.

The assessment of the risk of bias is shown in Fig. 2. The overall risk of bias was considered to be low in six studies^9, 12, 13, 18, 26, 28^, and high in one trial^27^. In that study, the bias arising from the randomization process was judged to be high^27^.

### Results of syntheses

#### Postoperative sore throat

**Figure 3:**
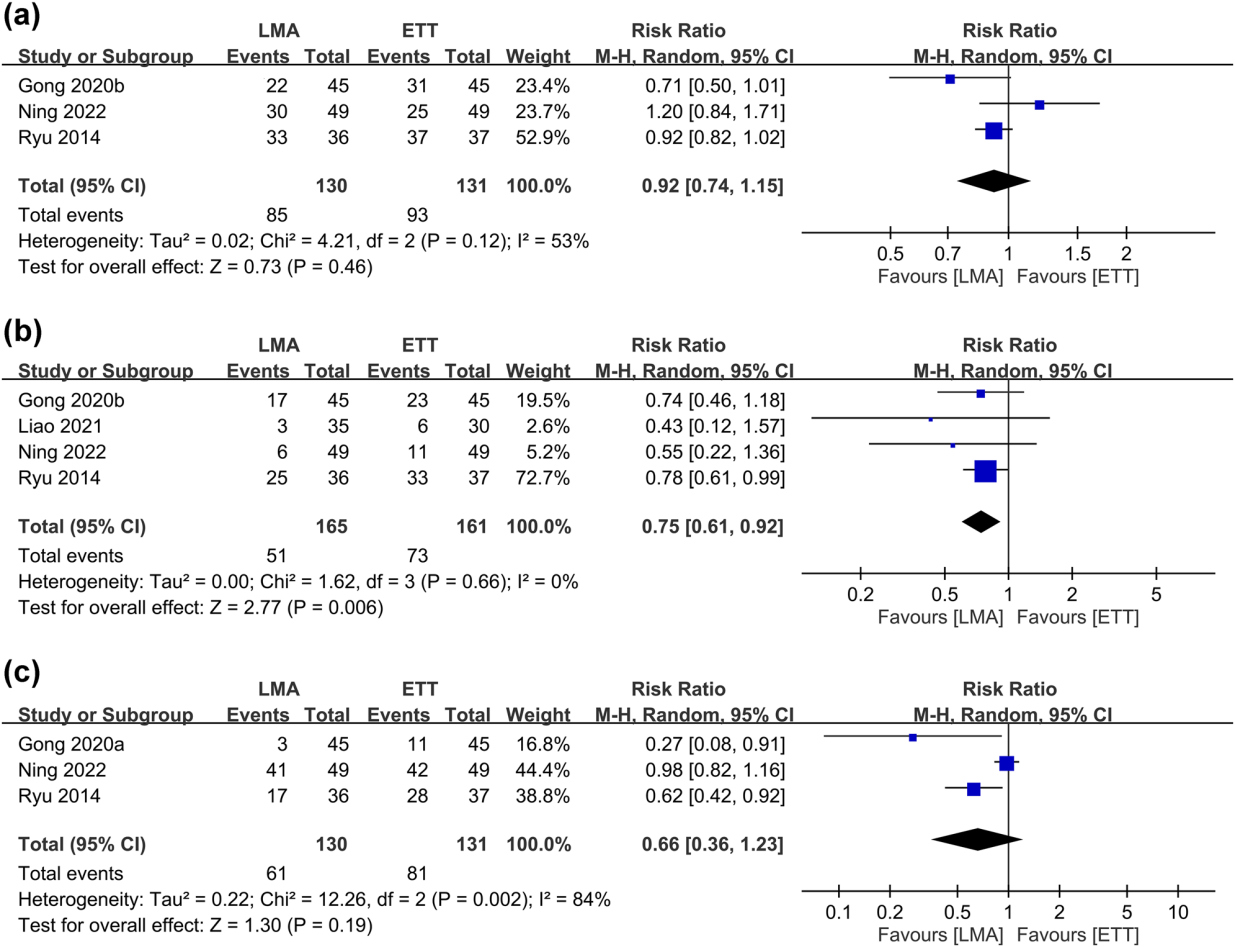
Forest plot comparing the risk of postoperative sore throat at (**a**) 1 h, (**b**) 24 h, and (**c**) 48 h between laryngeal mask airway (LMA) and endotracheal tube (ETT) groups. *CI* confidence interval, *M–H* Mantel–Haenszel.

**Figure 4:**
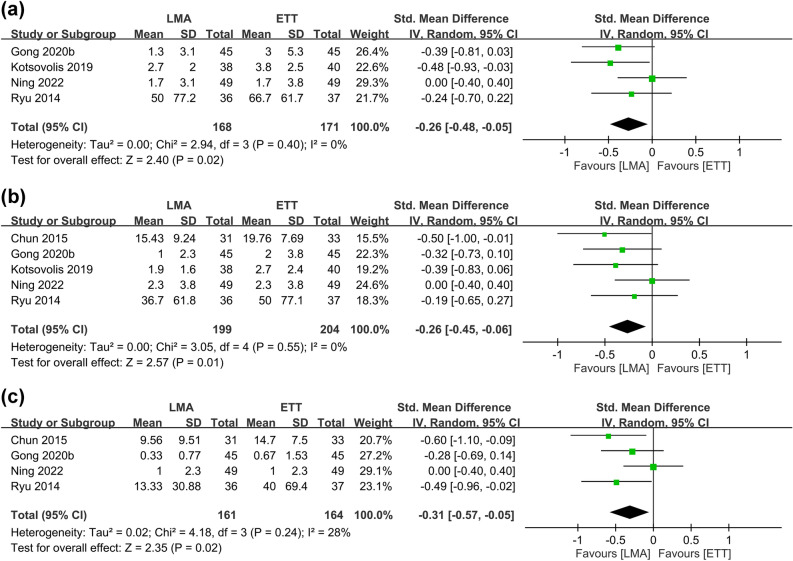
Forest plot comparing the severity of postoperative sore throat at (**a**) 1 h, (**b**) 24 h, and (**c**) 48 h between laryngeal mask airway (LMA) and endotracheal tube (ETT) groups. *CI* confidence interval, *IV* inverse variance, *Std* standardized.

The risks of POST at 1, 24, and 48 h are shown in Fig. 3^9, 13, 27, 28^. Meta-analysis revealed comparable risks of POST at one (RR = 0.92, 95% CI 0.74–1.15, *p* = 0.46, I^2^ = 53%, 261 patients) and 48 h (RR = 0.66, 95% CI 0.36–1.23, *p* = 0.19, I^2^ = 84%, 261 patients) between patients receiving LMA and those with ETT, while the former was associated with a lower risk of POST at 24 h (RR = 0.75, 95% CI 0.61–0.92, *p* = 0.006, I^2^ = 0%, 326 patients). Sensitivity analysis demonstrated stable merged results of the risk of POST. The severity of POST is shown in Fig. 4, which revealed a lower severity of POST in the LMA group compared to that in the ETT group at one, 12, and 24 h (Fig. 4). Nevertheless, sensitivity analysis demonstrated unstable results at the three time points.

#### Postoperative hoarseness

**Figure 5:**
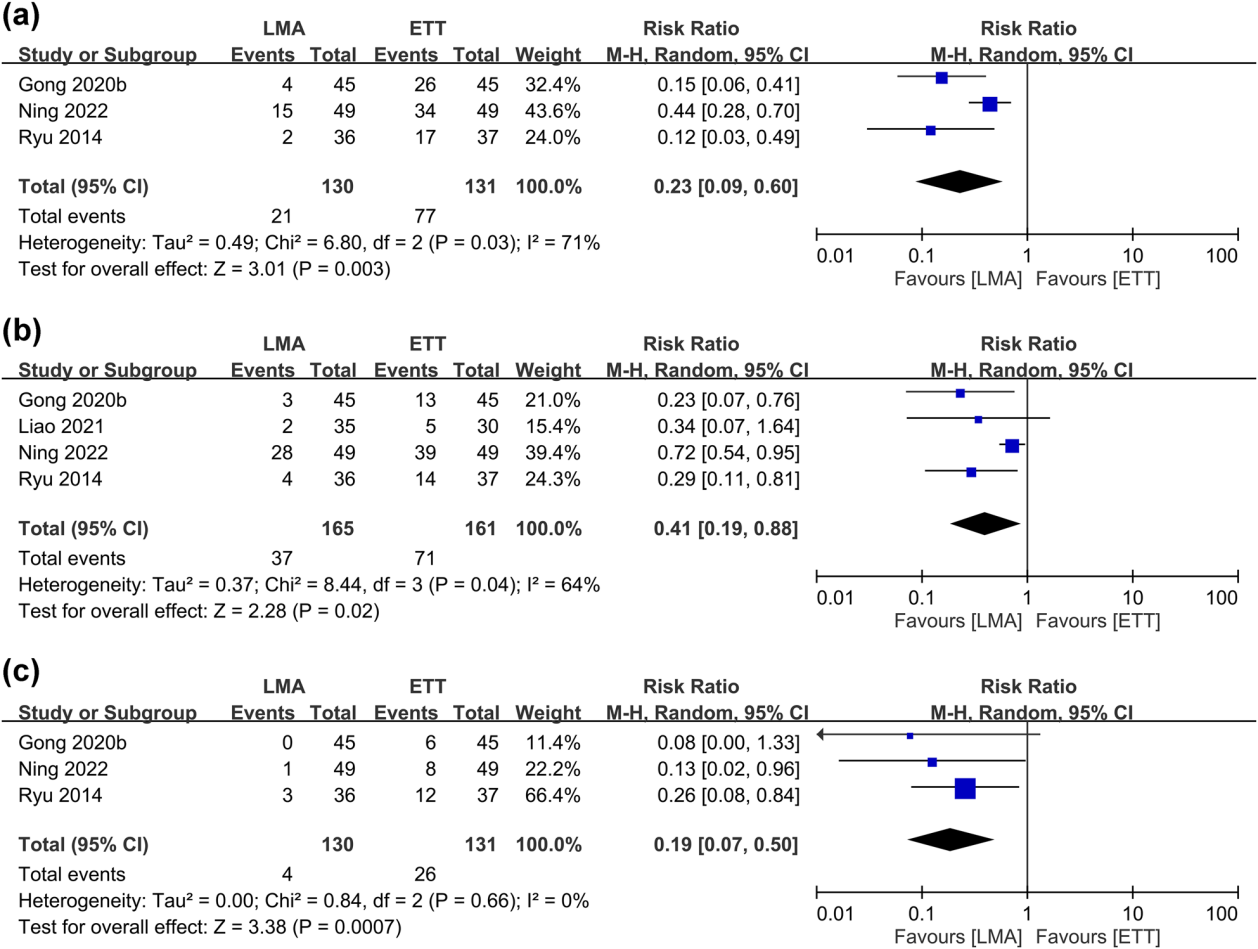
Forest plot comparing the risk of postoperative hoarseness at (**a**) 1 h, (**b**) 24 h, and (**c**) 48 h between laryngeal mask airway (LMA) and endotracheal tube (ETT) groups. *CI* confidence interval, *M–H* Mantel–Haenszel.

**Figure 6:**
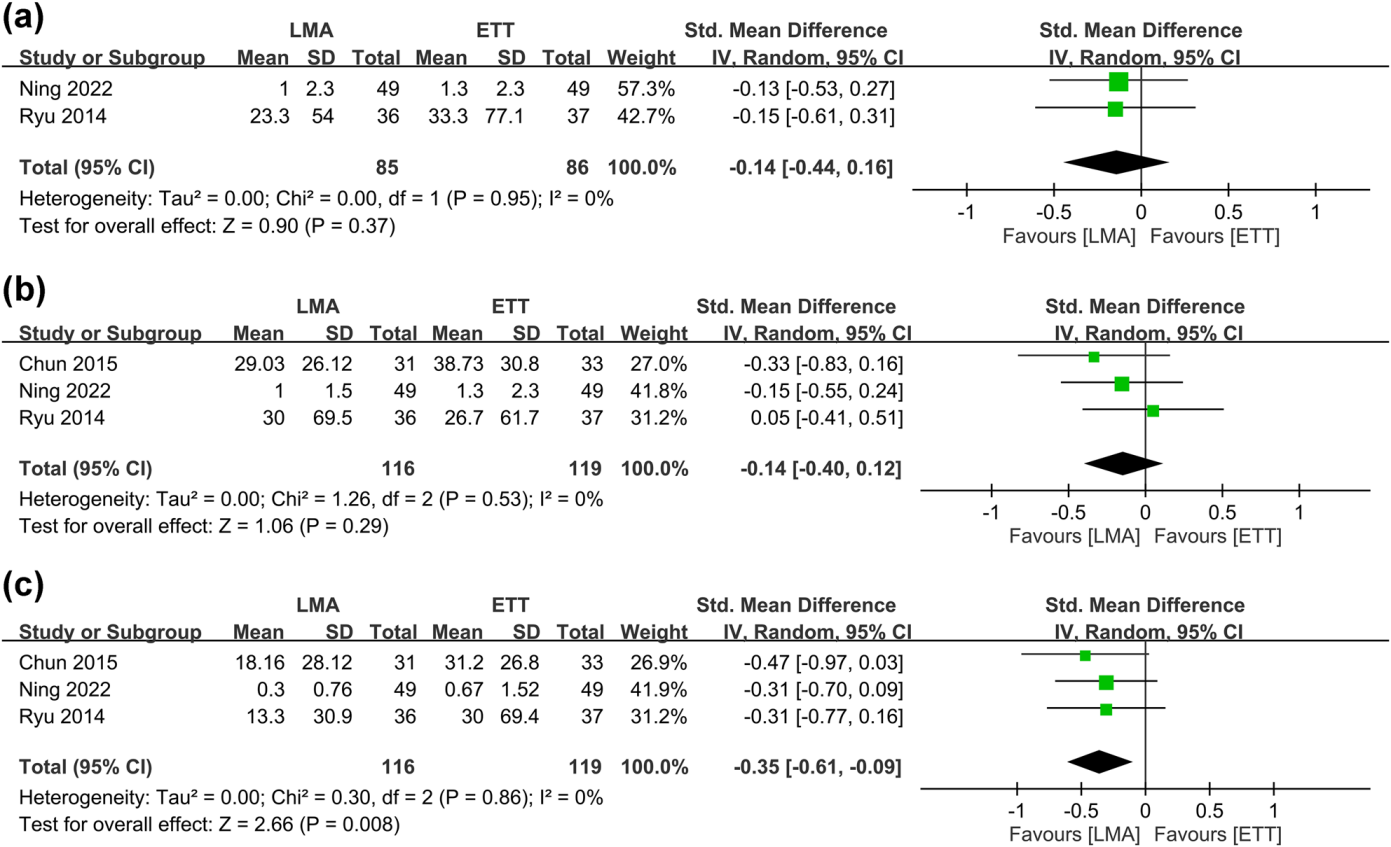
Forest plot comparing the severity of hoarseness at (**a**) 1 h, (**b**) 24 h, and (**c**) 48 h between laryngeal mask airway (LMA) and endotracheal tube (ETT) groups. *CI* confidence interval, *IV* inverse variance, *Std* standardized.

The risks of postoperative hoarseness at 1, 24, and 48 h, which were provided in four of the included studies, are detailed in Fig. 5^9, 13, 27, 28^. No nerve injury was reported in all of the seven included studies. Compared with ETT, the use of LMA was related to a lower risk of postoperative hoarseness at 1 h (RR = 0.23, 95% CI 0.09–0.6, *p* = 0.003, I^2^ = 71%, 261 patients), 24 h (RR = 0.41, 95% CI 0.19–0.88, *p* = 0.02, I^2^ = 64%, 326 patients), and 48 h (RR = 0.19, 95% CI 0.07–0.5, *p* = 0.0007, I^2^ = 0%, 261 patients) (Fig. 5). Through removing one study each time, we discovered instability of the merged results on the risk of hoarseness at postoperative one and 24 h. Analysis of the severity of hoarseness showed no differences between the two groups at one and 12 h, while it was lower in the LMA group at 48 h (SMD = − 0.35, 95% CI − 0.61 to − 0.09, *p* = 0.008, I^2^ = 0, 235 patients) (Fig. 6). Sensitivity analysis suggested instability of the results at 48 h.

#### Postoperative dysphagia, intraoperative airway pressure, and emergence cough

**Figure 7:**
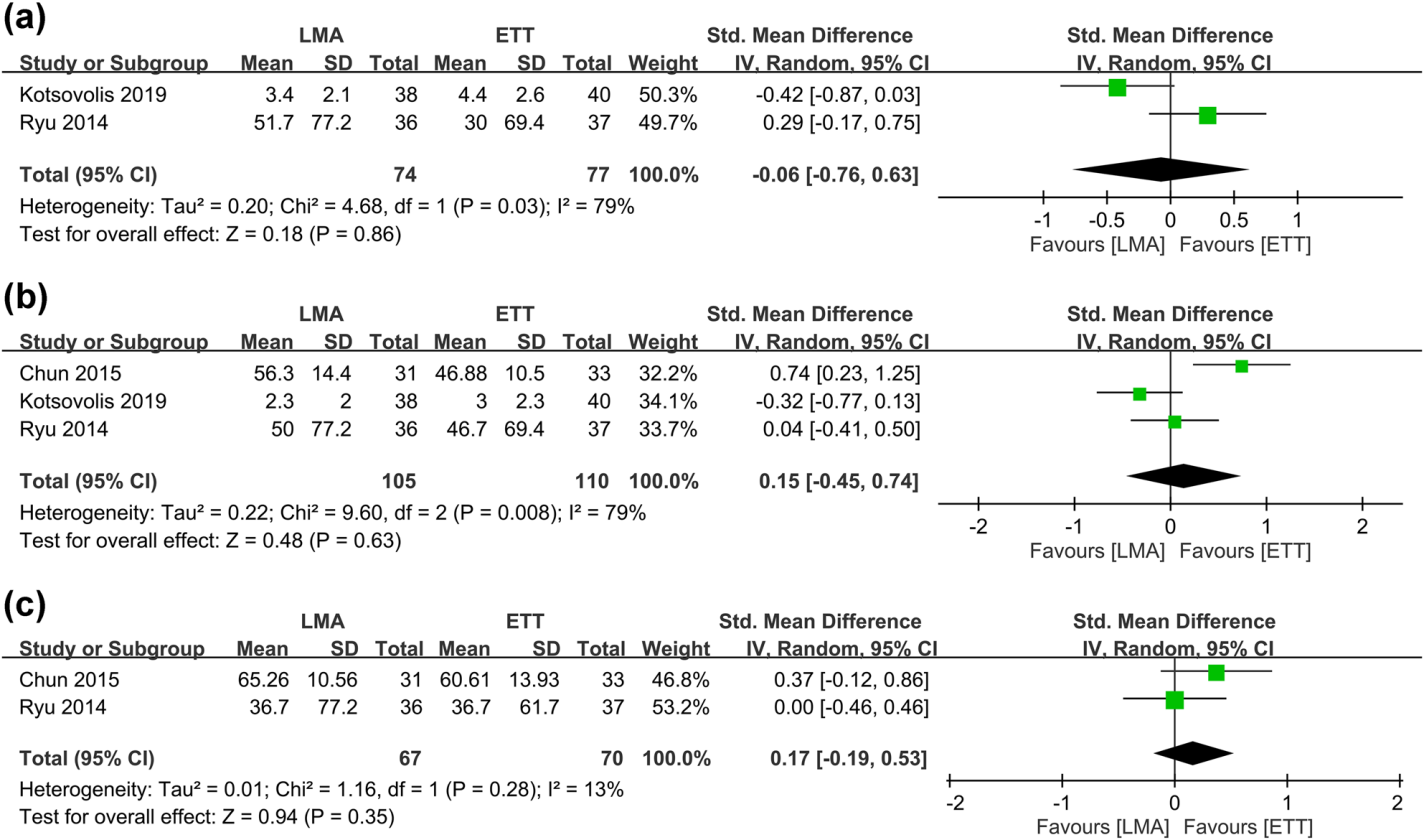
Forest plot comparing the severity of dysphagia at (**a**) 1 h, (**b**) 24 h, and (**c**) 48 h between laryngeal mask airway (LMA) and endotracheal tube (ETT) groups. *CI* confidence interval, *IV* inverse variance, *Std* standardized.

The severity of dysphagia in the both groups is available in Fig. 7^12, 13, 18^, which demonstrated no differences at 1, 24, and 48 h between the two groups. Sensitivity analysis also supported the stability of this finding.

**Figure 8:**
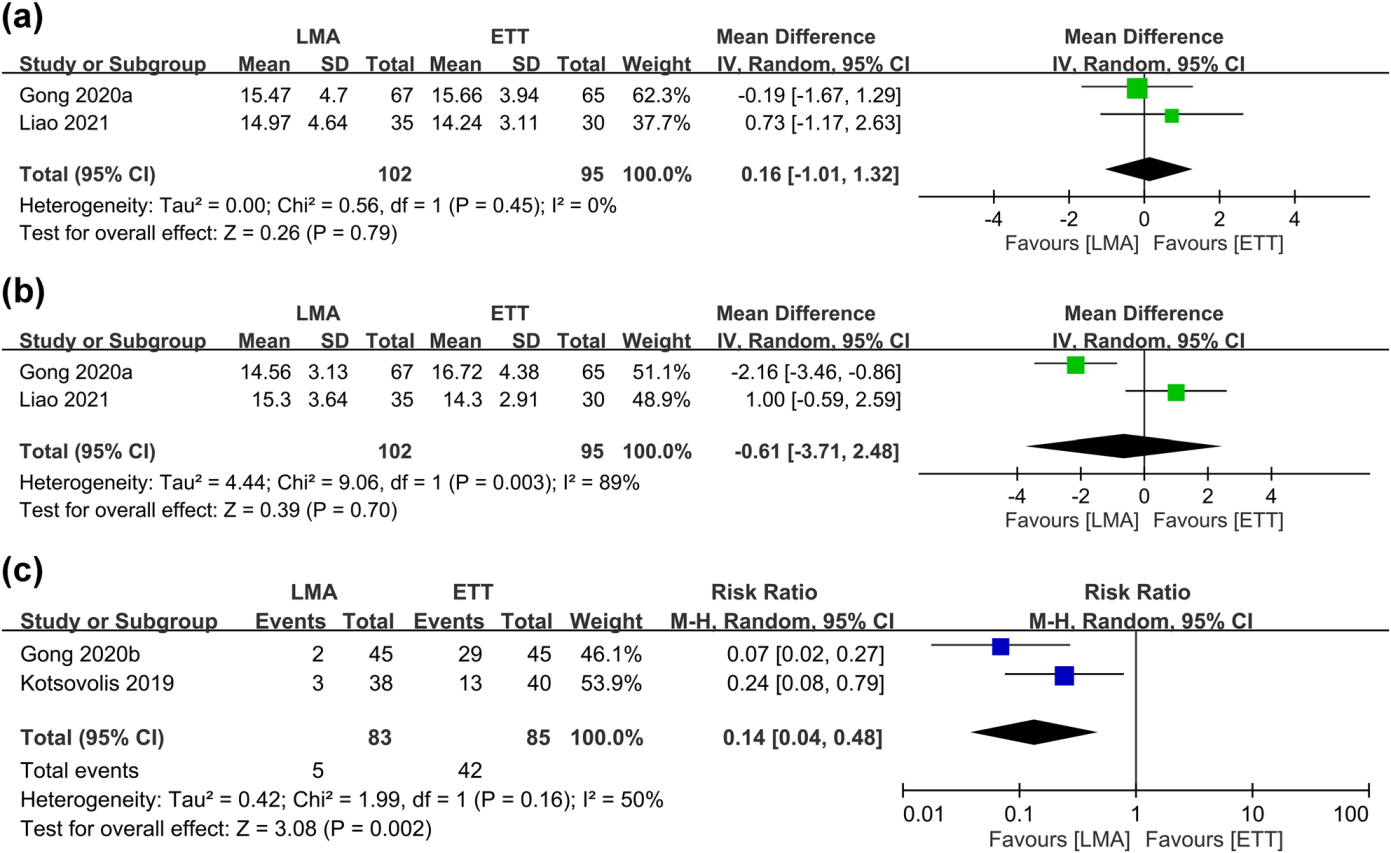
Forest plot comparing the differences in airway pressure at (**a**) 30 min and (**b**) 60 min after surgical incision as well as (**c**) risk of emergence cough between laryngeal mask airway (LMA) and endotracheal tube (ETT) groups. *CI* confidence interval, *IV* inverse variance, *Std* standardized, *M–H* Mantel–Haenszel.

Analysis of intraoperative airway pressure between the two groups showed no differences at 30 min (MD 0.16, 95% CI − 1.01 to 1.32 cm H_2_O, *p* = 0.79, I^2^ = 0, 197 participants (Fig. 8a) and 60 min (MD − 0.61, 95% CI − 3.71 to 2.48 cm H_2_O, *p* = 0.7, I^2^ = 89%, 197 participants) (Fig. 8b) after surgical incision. Sensitivity analysis showed unstable merged results at 60 min.

On the other hand, an investigation into the risk of emergence cough revealed a lower risk associated with LMA use compared to that with ETT (RR 0.14, 95% CI, 0.04–0.48, *p* = 0.002, I^2^ = 50%, 168 participants) (Fig. 8c). Sensitivity analysis demonstrated stability of the results.

#### Impact of LMA on quality of voice

Only one of the seven included studies performed acoustic analyses preoperatively and at postoperative 48 h and 2 weeks^12^. In that study^12^, acoustic analysis revealed significantly better improvements in jitter, shimmer, and noise-to-harmonic ratio in the LMA group than in the ETT group at 48 h after surgery, but without significant difference at 2 weeks. The incidence of postoperative lower-pitched voice in the former was also significantly lower than that in the latter.

The voice handicap index (VHI) was determined preoperatively and at post-thyroidectomy 24 and 48 h as well as one and two weeks. Despite postsurgical deterioration in VHI in both groups, patients with LMA exhibited a significantly better improvement compared to those receiving ETT at postoperative 24 h. While the former showed a persistent improvement to a degree comparable to their preoperative state at postoperative 1 week, the latter demonstrated improvement to their preoperative level only at 2 weeks after surgery.

## Discussion

Our results demonstrated that the use of LMA was associated with a reduced risk of POST at 24 h, but not at one and 48 h after thyroid surgery. Besides, the severity of POST and risk of postoperative hoarseness were lower in the LMA group compared to the ETT group at 1, 24, and 48 h. On the other hand, the severity of hoarseness was only found to be lower in the LMA group than in the ETT group only at postsurgical 48 h but not at other time points. In addition, the risk of emergence cough was lower in patients using LMA compared to those receiving ETT. There was no difference in airway pressure between the two groups at post-incision 30 and 60 min. Furthermore, there was no significant difference in the severity of dysphagia between the two groups at postoperative 1, 24, and 48 h.

Previous investigations have reported an association between laryngeal morbidity after tracheal intubation with endotracheal tube size as well as cuff design and pressure^5, 29–31^. Focusing on POST in adult patients receiving tracheal intubation, a previous study has identified the risk factors as female gender, younger age, pre-existing pulmonary disease, prolonged anesthesia, and the discovery of a blood-stained tracheal tube on extubation^2^. Moreover, the risk of POST could also be increased by tracheal intubation without neuromuscular blockade, the use of double-lumen tubes, and high tracheal tube cuff pressures^2^. The reported advantages of LMA over ETT include avoidance of the need for muscle relaxants and reversal as well as less hemodynamic response on insertion and removal and a reduced incidence of POST^12, 32–34^. Taking into account the finding of pharyngolaryngeal discomforts as the major complaints that contribute to distress and anxiety in patients following thyroid surgery^35^ as well as the growing number of thyroid operations being performed in an ambulatory or outpatient settings, the efficacy and safety of LMA use in this patient population has become an important clinical concern^36, 37^.

With regard to airway complications, a previous meta-analysis had demonstrated significantly lower incidences of postoperative hoarseness, coughing, and sore throat in LMA users compared to their ETT counterparts^15^. However, that meta-analytical study^15^ did not include the patient subgroup undergoing thyroid surgery. Our study, which is the first to investigate the impact of LMA on postoperative pharyngolaryngeal complications in patients undergoing thyroidectomy, showed favorable outcomes regarding the risks of POST at postoperative 24 h and hoarseness at one hour as well as 24 and 48 h after surgery. These findings are of clinical significance as outpatient thyroidectomy is increasingly preferred to the inpatient setting^38, 39^.

Even in the absence of nerve injury, patients undergoing thyroidectomy could experience postoperative voice dysfunction^40–42^ and as high as 25–90% of patients reported subjective voice alterations^43^. The most common complaint during objective and subjective evaluations is pitch-lowering^44^, the mechanism of which following uncomplicated thyroidectomy remains unclear^45^. Some authors proposed that the lower pitch voice could be due to a decrease in cordal tension resulting from functional alterations of the cricothyroid muscle^46^, while others attributed it to fibrosis from damage to the strap muscles that affects upward and/or downward laryngeal movement^47^. In the current meta-analysis, one of the included studies identified an association between the use of LMA and a lower incidence of postoperative lower-pitched voice than that in the ETT group^12^. The previous finding of a lower VHI in patients using LMA compared with those receiving ETT also supported the use of LMA in this clinical setting^12^. These findings may suggest the beneficial effect of LMA against voice dysfunction after thyroidectomy.

Nevertheless, one of the reported disadvantages of LMA compared to ETT was a lower sealing pressure and a higher incidence of gastric insufflation associated with the former^32^. Despite the previous finding that the performance of LMA may be affected by changes in head and neck position that unavoidably alter the pharyngeal structures^48, 49^, a previous meta-analysis demonstrated no correlation between an extended neck position and an impairment in ventilation in most type of LMA^50^. Furthermore, previous evidence showed no difference in the incidence of desaturation, regurgitation, gastric insufflations, and pulmonary aspiration between LMA and ETT in the general population^15, 51, 52^. On the other hand, there are still concerns over the safety of LMA use in patients receiving thyroid surgery^53^ due to the potential dangers of intraoperative dislodgment and inadequate ventilation that could not be timely corrected through emergent airway access because of potential interference with ongoing surgery^26^. However, previous investigations have demonstrated a rarity of intraoperative conversion from LMA to ETT^12, 54^ and one of our included studies showed that mild to moderate shift of the LMA during surgery was insufficient to impede ventilation^26^.

Another safety concern associated with the use of LMA is potential fluctuations in airway pressure. Despite the absence of difference in airway pressure at postoperative 30 and 60 min between LMA and ETT in the current meta-analysis, one of our included trials reported markedly elevated peak airway pressure through tracheal angulation and laryngeal rotation from forceful intraoperative retraction of thyroid tissue^26^. The authors of that study recommended effective communication with the surgeons with adjustment of the operating table to reestablish adequate ventilation^26^.

The current meta-analysis had its limitations. First, because our included trials excluded certain patient subgroups including those with obesity^9, 13, 26^, recent upper airway infection^9, 12, 13, 26, 28^, and difficult airways^12, 18, 26, 27^ as well as those having received oropharyngeal surgeries^9, 13, 26, 28^, our findings may not be applicable to these patient populations. Second, the incidence and severity of POST have been shown to vary with different LMAs^55^ with the LMA i-gel being reported to be associated with the lowest incidence of POST compared to the others^56^. Nevertheless, the limited number of studies in current meta-analysis precluded a subgroup analysis of such an impact. Third, inconsistency of our findings over different time points may be attributable to the small number of included studies. Further large-scale investigation is needed to support our findings.

## Conclusion

The current meta-analysis showed an association between the use of LMA and a reduced risk of POST at 24 h as well as a lower severity of POST and risk of postoperative hoarseness in the LMA group compared to the patients receiving ETT at one, 24, and 48 h. Besides, the risk of emergence cough was lower in patients using LMA than those receiving ETT. Compared with ETT, our results supported the use of LMA in patients undergoing thyroid surgery in minimizing postoperative POST and hoarseness without causing significant increases in the severity of dysphagia or fluctuation in airway pressure. Further large-scale studies are warranted to verify our findings.

## Supplementary Information

Supplementary Information.

## Data Availability

The datasets used and/or analysed during the current study available from the corresponding author on reasonable request.

## References

[1] Higgins PP, Chung F, Mezei G (2002). Postoperative sore throat after ambulatory surgery. BJA Br. J. Anaesth..

[2] El-Boghdadly K, Bailey CR, Wiles MD (2016). Postoperative sore throat: A systematic review. Anaesthesia.

[3] Macario A, Weinger M, Carney S, Kim A (1999). Which clinical anesthesia outcomes are important to avoid? The perspective of patients. Anesth. Analg..

[4] Lehmann M, Monte K, Barach P, Kindler CH (2010). Postoperative patient complaints: A prospective interview study of 12,276 patients. J. Clin. Anesth..

[5] McHardy FE, Chung F (1999). Postoperative sore throat: Cause, prevention and treatment. Anaesthesia.

[6] Martis C, Athanassiades S (1971). Post-thyroidectomy laryngeal edema. A survey of fifty-four cases. Am. J. Surg..

[7] Hisham AN, Roshilla H, Amri N, Aina EN (2001). Post-thyroidectomy sore throat following endotracheal intubation. ANZ J. Surg..

[8] Nouraei S, Virk J, Middleton S (2017). A national analysis of trends, outcomes and volume–outcome relationships in thyroid surgery. Clin. Otolaryngol..

[9] Gong Y, Xu X, Wang J, Che L, Wang W, Yi J (2020). Laryngeal mask airway reduces incidence of post-operative sore throat after thyroid surgery compared with endotracheal tube: A single-blinded randomized controlled trial. BMC Anesthesiol..

[10] Joshi GP, Inagaki Y, White PF (1997). Use of the laryngeal mask airway as an alternative to the tracheal tube during ambulatory anesthesia. Anesth. Analg..

[11] Martin-Castro C, Montero A (2008). Flexible laryngeal mask as an alternative to reinforced tracheal tube for upper chest, head and neck oncoplastic surgery. Eur. J. Anaesthesiol..

[12] Chun BJ, Bae JS, Lee SH, Joo J, Kim ES, Sun DI (2015). A prospective randomized controlled trial of the laryngeal mask airway versus the endotracheal intubation in the thyroid surgery: Evaluation of postoperative voice, and laryngopharyngeal symptom. World J. Surg..

[13] Ryu JH, Yom CK, Park DJ (2014). Prospective randomized controlled trial on the use of flexible reinforced laryngeal mask airway (LMA) during total thyroidectomy: Effects on postoperative laryngopharyngeal symptoms. World J. Surg..

[14] Jefferson N, Riffat F, McGuinness J, Johnstone C (2011). The laryngeal mask airway and otorhinolaryngology head and neck surgery. Laryngoscope.

[15] Yu SH, Beirne OR (2010). Laryngeal mask airways have a lower risk of airway complications compared with endotracheal intubation: A systematic review. J. Oral Maxillofac. Surg..

[16] Radu AD, Miled F, Marret E, Vigneau A, Bonnet F (2008). Pharyngo-laryngeal discomfort after breast surgery: Comparison between orotracheal intubation and laryngeal mask. Breast (Edinburgh, Scotland).

[17] Bennett J, Petito A, Zandsberg S (1996). Use of the laryngeal mask airway in oral and maxillofacial surgery. J. Oral Maxillofac. Surg..

[18] Kotsovolis G, Pliakos I, Panidis S, Gkinas D, Papavramidis T (2019). Comparison between the protector TM Laryngeal mask airway and the endotracheal tube for minimally invasive thyroid and parathyroid surgery. World J. Surg..

[19] Sterne JAC, Savović J, Page MJ (2019). RoB 2: A revised tool for assessing risk of bias in randomised trials. BMJ (Clin. Res. Ed.).

[20] Hung KC, Chu CC, Hsing CH (2021). Association between perioperative intravenous lidocaine and subjective quality of recovery: A meta-analysis of randomized controlled trials. J. Clin. Anesth..

[21] Hung KC, Ho CN, Chen IW (2021). The impact of aminophylline on incidence and severity of post-dural puncture headache: A meta-analysis of randomised controlled trials. Anaesth. Crit. Care Pain Med..

[22] Wan X, Wang W, Liu J, Tong T (2014). Estimating the sample mean and standard deviation from the sample size, median, range and/or interquartile range. BMC Med. Res. Methodol..

[23] Hung KC, Chang YJ, Chen IW (2022). Efficacy of high flow nasal oxygenation against hypoxemia in sedated patients receiving gastrointestinal endoscopic procedures: A systematic review and meta-analysis. J. Clin. Anesth..

[24] Hung KC, Chiang MH, Wu SC (2021). A meta-analysis of randomized clinical trials on the impact of oral vitamin C supplementation on first-year outcomes in orthopedic patients. Sci. Rep..

[25] Hung KC, Chen JY, Feng IJ (2021). Efficacy and airway complications of Parker Flex-Tip tubes and standard endotracheal tubes during airway manipulation: A meta-analysis and trial sequential analysis. Eur. J. Anaesthesiol..

[26] Gong Y, Wang J, Xu X, Li J, Song R, Yi J (2020). Performance of air seal of flexible reinforced laryngeal mask airway in thyroid surgery compared with endotracheal tube: A randomized controlled trial. Anesth. Analg..

[27] Liao H, Chen L, Sheng C (2021). The effects of on hemodynamics, oxygen saturation, peak airway pressure and adverse events during anesthesia for thyroid surgery: tracheal intubation Vs. ProSeal laryngeal mask airway. Am. J. Transl. Res..

[28] Ning M, Zhong W, Li J, Wang T, Lu Y (2022). Comparison between I-gel(®) and endotracheal intubation in terms of the incidence of postoperative sore throat following thyroid surgery: A randomized observational trial. Am. J. Transl. Res..

[29] Biro P, Seifert B, Pasch T (2005). Complaints of sore throat after tracheal intubation: A prospective evaluation. Eur. J. Anaesthesiol..

[30] Combes X, Schauvliege F, Peyrouset O (2001). Intracuff pressure and tracheal morbidity: Influence of filling with saline during nitrous oxide anesthesia. Anesthesiology.

[31] Mencke T, Echternach M, Kleinschmidt S (2003). Laryngeal morbidity and quality of tracheal intubation: A randomized controlled trial. Anesthesiology.

[32] Brimacombe J (1995). The advantages of the LMA over the tracheal tube or facemask: A meta-analysis. Can. J. Anaesth..

[33] Soylu L, Ozbas S, Uslu HY, Kocak S (2007). The evaluation of the causes of subjective voice disturbances after thyroid surgery. Am. J. Surg..

[34] Yaman F, Arslan B, Yuvanç E, Büyükkoçak U (2014). Unexpected difficult airway with hypogonadotropic hypogonadism. Int. Med. Case Rep. J..

[35] Jung TH, Rho JH, Hwang JH, Lee JH, Cha SC, Woo SC (2011). The effect of the humidifier on sore throat and cough after thyroidectomy. Korean J. Anesthesiol..

[36] Frank E, Park J, Simental A (2017). Six-year experience of outpatient total and completion thyroidectomy at a single academic institution. Am. Surg..

[37] Lee DJ, Chin CJ, Hong CJ, Perera S, Witterick IJ (2018). Outpatient versus inpatient thyroidectomy: A systematic review and meta-analysis. Head Neck.

[38] McLaughlin EJ, Brant JA, Bur AM (2018). Safety of outpatient thyroidectomy: Review of the American College of Surgeons National Surgical Quality Improvement Program. Laryngoscope.

[39] van Gerwen M, Alsen M, Alpert N, Sinclair C, Taioli E (2022). Trends for in- and outpatient thyroid cancer surgery in older adults in New York State, 2007–2017. J. Surg. Res..

[40] Aluffi P, Policarpo M, Cherovac C, Olina M, Dosdegani R, Pia F (2001). Post-thyroidectomy superior laryngeal nerve injury. Eur. Arch. Oto-rhino-laryngol..

[41] Beka, E. & Gimm, O. Voice changes without laryngeal nerve alterations after thyroidectomy: The need for prospective trials—A review study. *J. Voice.* (2021). 10.1016/j.jvoice.2021.07.012. 10.1016/j.jvoice.2021.07.012 34404582

[42] McIvor NP, Flint DJ, Gillibrand J, Morton RP (2000). Thyroid surgery and voice-related outcomes. Aust. N. Z. J. Surg..

[43] Henry LR, Solomon NP, Howard R (2008). The functional impact on voice of sternothyroid muscle division during thyroidectomy. Ann. Surg. Oncol..

[44] Van Lierde K, D'Haeseleer E, Wuyts FL, Baudonck N, Bernaert L, Vermeersch H (2010). Impact of thyroidectomy without laryngeal nerve injury on vocal quality characteristics: An objective multiparameter approach. Laryngoscope.

[45] Nam IC, Park YH (2017). Pharyngolaryngeal symptoms associated with thyroid disease. Curr. Opin. Otolaryngol. Head Neck Surg..

[46] Nam IC, Bae JS, Chae BJ, Shim MR, Hwang YS, Sun DI (2013). Therapeutic approach to patients with a lower-pitched voice after thyroidectomy. World J. Surg..

[47] Hong KH, Ye M, Kim YM, Kevorkian KF, Berke GS (1997). The role of strap muscles in phonation—in vivo canine laryngeal model. J. Voice.

[48] Kim HJ, Lee K, Bai S, Kim MH, Oh E, Yoo YC (2017). Influence of head and neck position on ventilation using the air-Q^®^ SP airway in anaesthetized paralysed patients: A prospective randomized crossover study. Br. J. Anaesth..

[49] Somri M, Vaida S, Garcia Fornari G (2016). A randomized prospective controlled trial comparing the laryngeal tube suction disposable and the supreme laryngeal mask airway: The influence of head and neck position on oropharyngeal seal pressure. BMC Anesthesiol..

[50] Kim MS, Park JH, Lee KY (2019). Influence of head and neck position on the performance of supraglottic airway devices: A systematic review and meta-analysis. PLoS ONE.

[51] Brimacombe JR, Berry A (1995). The incidence of aspiration associated with the laryngeal mask airway: A meta-analysis of published literature. J. Clin. Anesth..

[52] Park SK, Ko G, Choi GJ, Ahn EJ, Kang H (2016). Comparison between supraglottic airway devices and endotracheal tubes in patients undergoing laparoscopic surgery: A systematic review and meta-analysis. Medicine.

[53] Xu R, Lian Y, Li WX (2016). Airway complications during and after general anesthesia: A comparison, systematic review and meta-analysis of using flexible laryngeal mask airways and endotracheal tubes. PLoS One.

[54] Pott L, Swick JT, Stack BC (2007). Assessment of recurrent laryngeal nerve during thyroid surgery with laryngeal mask airway. Arch. Otolaryngol. Head Neck Surg..

[55] Lin GJW, Lim YC, Wang J, Shahla S (2020). An audit of post-operative sore throat using different laryngeal mask airways. Indian J. Anaesth..

[56] Russo SG, Cremer S, Galli T (2012). Randomized comparison of the i-gel™, the LMA Supreme™, and the Laryngeal Tube Suction-D using clinical and fibreoptic assessments in elective patients. BMC Anesthesiol..

